# Supplementary material for: Evaluating Alternatives to Water as Solvents for Life: The Example of Sulfuric Acid
Source: Life (Basel). 2021 Apr 27;11(5):400. doi: 10.3390/life11050400 (PMC8145300; doi:10.3390/life11050400)
Supplement: Supplementary file 1 [file life-11-00400-s001.zip › Bains et al Supplementary File S1.pdf]

A framework for evaluation of potential solvents for life

We describe a simple framework for evaluating a potential solvent as a basis for biochemistry. Our purpose in laying out these criteria is to provide a basis on which to move beyond qualitative statements to a semi-quantitative evaluation of the plausibility of a solvent as the basis of life. If terrestrial life is typical of life elsewhere (an assumption we are forced to make for lack of any other), then the solvent for life needs to have two key properties. The solvent has to be able to dissolve some (but not all) molecules, and some (but not all) molecules, including some that it dissolves, must be stable in its presence [4].

Both stability and solvation have to allow for the existence of a diverse chemical space to provide the chemical functionality needed for life. "Chemical space" as a concept is well established [5,6], in which relevant properties of a large set of compounds are used to map them onto a space that can be used to predict their properties or function (e.g. [7,8]). In this paper we wish to explore whether there is a subset of all possible chemicals that is stable and soluble in sulfuric acid and sufficiently extensive to be a candidate for a biochemistry. We use two proxies for the chemical space of 'all possible chemicals' – a diverse space of actual compounds made both my life and by human ingenuity, supplemented with a small fraction of the space of theoretical structures that follow basic bonding rules.

Solvation and chemical stability criteria

Solvation. The solvent needs to be able to dissolve a wide range of molecules, including macromolecules, and to not dissolve other molecules. The need for solvation of some molecules is obvious [4,9]; rapid chemical reactions only occur between molecules in solution or in gas phase, and gas phases cannot dissolve macromolecules. The need for insolubility of some molecules comes from two requirements. First, the structural elements (e.g. cellulose in plants on Earth) must be essentially insoluble in their surrounding solvent if they are to function as cellular barriers and stable supports. Second, insolubility allows for the existence of molecules in which part of the molecule is highly soluble and another part of the molecule is insoluble in the solvent [10]. Such characteristics allows for the assembly of nanostructures such as membranes and globular proteins [11], driven by solvation properties. Thus for example liquid methane, despite being sometimes considered as a possible solvent for life [1,12-15], is an unlikely solvent for life because very few molecules dissolve in it [16]. Water is highly favored as a solvent for life because many molecules are highly insoluble in water, allowing for a 'hydrophobic' force that drives the folding of proteins, assembly and stability of membranes etc. [10,17]. We note that in the case of water the 'hydrophobic' force is driven by entropic effects of solvating a non-hydrogen bonding molecule into the liquid water structure. However other thermodynamic drivers could work in apolar solvents, as suggested by [1-3].

Stability. The solvent needs to allow the stable solution of a diverse set of chemicals. Thus silicate magma is not a plausible solvent for life [18] because very few organic molecules are stable at the melting temperature of mantle silicates. The chemistry of life requires molecules that differ in chemical stability at ambient temperature. The need for a diverse set of chemicals that have a wide spectrum of chemical stabilities is illustrated by the requirement of molecules that act as reaction intermediates in metabolism. Reaction intermediates with excessive stability make evolution of efficient catalysts for reactions involving such intermediates exceedingly difficult. The case of elemental nitrogen (N_2) illustrates this point. While in principle the reduction of N_2 by NADH to form NH_3 is highly exergonic, the extreme strength of the nitrogen-nitrogen triple bond requires life to use an additional 8 moles of ATP per mole of NH_3 formed to overcome the activation energy of this reaction [19]. It is not plausible that life would expend this amount of energy on every reaction in metabolism. By contrast, ATP is stable in aqueous solution, but the kinetic barrier to its hydrolysis is relatively small, as illustrated by its rapid spontaneous hydrolysis at >200 ^{o}C [20,21]. Thus ATP is widely used as an energy carrier, whereas the ability to metabolize N_2 is limited. The solvent may play a role in the relative stability of metabolites; if the solvent is chemically inert, the requirement for a range of stabilities places additional constraints on the chemistry from which biochemistry can be constructed.

Other physical properties of the solvent, e.g. viscosity, may also be relevant for the solvent to be conducive to life. The solvent must have a low enough viscosity to allow large molecules to diffuse through it and interact. Thus, for example, bitumen is technically a liquid, but its viscosity is so high at Earth surface temperatures that it is an implausible solvent for life. [22] also suggest that too low a viscosity is incompatible with the non-equilibrium thermodynamics of macromolecular catalysis.

Solvation, viscosity and stability are all strongly temperature dependent, and so whether a substance is a suitable solvent for life depends on its temperature. Pressure has little effect at the pressures likely to apply to rocky planet surfaces or atmospheres [23].

Water is an essential reagent in terrestrial biochemistry, and as such water is not just a solvent but is a participant in biochemistry. It is widely stated that water's chemical properties are important in the chemistry of life, and specifically for selectivity at the origin of life [4,24]. Moreover, water's ability to solvate protons and hydroxyl ions is central to a wide range of biochemistry, including proton gradient-based bioenergetics. We are unconvinced that this limits the selection of a solvent for life. If a chemical group commonly used by life is not present in the solvent, and is not soluble or stable in the solvent, then biochemistry can mitigate this by the use of carrier molecules. Thus electrons are not stable when dissolved in water, so terrestrial life uses a wide range of electron carrier molecules to transport electrons between molecules in redox reactions. Water spontaneously ionizes to form H_3O^+ and OH^- ions, and life makes extensive use of gradients of H_3O^+ ions in energy metabolism. Dry ammonia does not ionize to the same degree (K_{eq} of the reaction $NH_3 \leftrightarrow NH_{4+} + NH_{2^-}$ is ~10^{-33} at 220 K [25]). In dry ammonia proton gradients are therefore less likely, but electron gradients could be formed as electrons can stably dissolve in ammonia [25]. In an apolar solvent, life could use –OH carriers to carry out hydration or dehydration reactions without generating highly insoluble water molecules. Obviously these ideas are completely speculative, but they illustrate that chemical arguments based on water's participation in the metabolism of life (as opposed to structural arguments above) are hard to defend on an absolute *a priori* basis.

Both stability and solubility criteria also have to apply to polymers, which need to show a range of stability and solubility properties. In water, for example, RNA is soluble enabling its role as an information intermediary in cells, whereas cellulose is not, enabling its role as a structural element.

Chemical diversity criteria

The solvent criteria discussed in Section 2.1. need to be applicable to a diverse set of molecules. Life needs to be able to access a diverse space of possible chemicals. A sufficiently chemically rich chemical space enables diverse chemical effects and allows for specific interactions between molecules [4]. The former requires a diverse set of chemical 'groups' (clusters of atoms bonded together with characteristic chemical properties), the latter requires diverse arrangements of atoms in space (topological structure) [4,16].

1. Stevenson, J.; Lunine, J.; Clancy, P. Membrane alternatives in worlds without oxygen: Creation of an azotosome. *Science Advances* **2015**, *1*, e1400067, doi:10.1126/sciadv.1400067.
2. Facchin, M.; Scarso, A.; Selva, M.; Perosa, A.; Riello, P. Towards life in hydrocarbons: Aggregation behaviour of "reverse" surfactants in cyclohexane. *RSC advances* **2017**, *7*, 15337-15341.
3. Sandström, H.; Rahm, M. Can polarity-inverted membranes self-assemble on Titan? *Science Advances* **2020**, *6*, eaax0272, doi:10.1126/sciadv.aax0272.
4. Hoehler, T.; Bains, W.; Davila, A.; Parenteau, M.; Pohorille, A. Life's requirements, habitability, and biological potential. *Planetary Astrobiology* **2020**, 37.

5. Kirkpatrick, P.; Ellis, C. Chemical space. *Nature* **2004**, *432*, 823-823, doi:10.1038/432823a.
6. Dobson, C.M. Chemical space and biology. Nature Publishing Group: 2004.
7. Reymond, J.-L.; Van Deursen, R.; Blum, L.C.; Ruddigkeit, L. Chemical space as a source for new drugs. *MedChemComm* **2010**, *1*, 30-38.
8. van Deursen, R.; Reymond, J.L. Chemical space travel. *ChemMedChem: Chemistry Enabling Drug Discovery* **2007**, *2*, 636-640.
9. Bains, W. Many chemistries could be used to build living systems. *Astrobiology* **2004**, *4*, 137-167.
10. Tanford, C. The hydrophobic effect and the organization of living matter. *Science* **1978**, *200*, 1012-1018.
11. Dill, K.A. Dominant forces in protein folding. *Biochemistry* **1990**, *29*, 7133-7155.
12. McKay, C.; Smith, H. Possibilities for methanogenic life in liquid methane on the surface of Titan. *Icarus* **2005**, *178*, 274-276.
13. McKay, C.P. Titan as the abode of life. *Life* **2016**, *6*, 8.
14. McLendon, C.; Opalko, F.J.; Illangkoon, H.I.; Benner, S.A. Solubility of polyethers in hydrocarbons at low temperatures. A model for potential genetic backbones on warm titans. *Astrobiology* **2015**, *15*, 200-206.
15. Norman, L.H. Is there life on… Titan? *Astronomy & Geophysics* **2011**, *52*, 1.39-31.42.
16. Petkowski, J.J.; Bains, W.; Seager, S. On the Potential of Silicon as a Building Block for Life. *Life* **2020**, *10*, 84.
17. Pohorille, A.; Pratt, L.R. Is Water the Universal Solvent for Life? *Origins of Life and Evolution of Biospheres* **2012**, *42*, 405-409, doi:10.1007/s11084-012-9301-6.
18. Feinberg, G.; Shapiro, R. Life beyond earth: the intelligent earthling's guide to life in the universe. *Life beyond earth: the intelligent earthling's guide to life in the universe* **1980**.
19. Kim, J.; Rees, D.C. Nitrogenase and biological nitrogen fixation. *Biochemistry* **1994**, *33*, 389-397.
20. Kunio, K. Monitoring Hydrothermal Reactions on the Millisecond Time Scale Using a Micro-Tube Flow Reactor and Kinetics of ATP Hydrolysis for the RNA World Hypothesis. *Bulletin of the Chemical Society of Japan* **2000**, *73*, 1805-1811, doi:10.1246/bcsj.73.1805.
21. Daniel, R.M.; Van Eckert, R.; Holden, J.F.; Truter, J.; Crowan, D.A. The stability of biomolecules and the implications for life at high temperatures. *GMS* **2004**, *144*, 25-39.
22. Branscomb, E.; Russell, M.J. On the beneficent thickness of water. *Interface Focus* **2019**, *9*, 20190061, doi:doi:10.1098/rsfs.2019.0061.
23. Bains, W.; Xiao, Y.; Yu, C. Prediction of the maximum temperature for life based on the stability of metabolites to decomposition in water. *Life* **2015**, *5*, 1054-1100.
24. council, N.R. The limits of organic life in planetary systems. The national academy of press: 2019.
25. Greenwood, N.N.; Earnshaw, A. *Chemistry of the Elements (2nd Edn)*; Elsevier: Amsterdam, 1997.
